# Long story short

**DOI:** 10.7554/eLife.20314

**Published:** 2016-09-06

**Authors:** John R Pannell, Guillaume Cossard

**Affiliations:** Department of Ecology and Evolution, University of Lausanne, Lausanne, SwitzerlandJohn.Pannell@unil.ch; Department of Ecology and Evolution, University of Lausanne, Lausanne, Switzerland

**Keywords:** heterostyly, Primula, brassinosteroids, supergene, S-locus, Other

## Abstract

One of the genes responsible for producing different "morphs" of primrose flowers has been identified.

**Related research article** Huu CN, Kappel C, Keller B, Sicard A, Takebayashi Y, Breuninger H, Nowak MD, Bäurle I, Himmelbach A, Burkart M, Ebbing-Lohaus T, Sakakibara H, Altschmied L, Conti E, Lenhard M. 2016. Presence versus absence of *CYP734A50* underlies the style-length dimorphism in primroses. *eLife* **5**:e17956. doi: 10.7554/eLife.17956

The vast majority of flowering plants are hermaphrodites, with both male and female parts in each of their flowers (Renner, 2014). This strategy is clearly a successful one, coming with several likely advantages including that, by producing both pollen and ovules, each flower has more opportunities to pass genes on to the next generation (Charnov et al., 1976). Although hermaphrodites might also benefit from the possibility of self-fertilization (and many do), most hermaphrodites do all they can to avoid ‘selfing’ because it is often detrimental to a plant’s fitness (Charlesworth and Willis, 2009; Barrett, 2002). Hermaphroditic plants have thus evolved a wonderful array of mechanisms that help them to promote outcrossing.

One of these mechanisms, known as heterostyly, involves male and female parts of the flower (the stamen and the stigma) being in different places in different flowers. The stigma sits on the end of a stalk called the style. Some flowers have long styles and hold their stamens, which produce pollen, deep in the floral tube, whereas others have short styles and hold their stamens much higher in the floral tube (Figure 1A). This means that pollen from a long-styled plant is more likely to pollinate a short-styled flower, and vice versa. Long- and short-styled plants may also differ in the size and colour of their pollen grains and the texture of their stigmas, and outcrossing is only possible between different morphs (Barrett, 2002). Remarkably, despite its complexity, heterostyly has evolved repeatedly in flowering plants and is found in at least 30 families, including the primroses (genus *Primula*).

**Figure 1.**
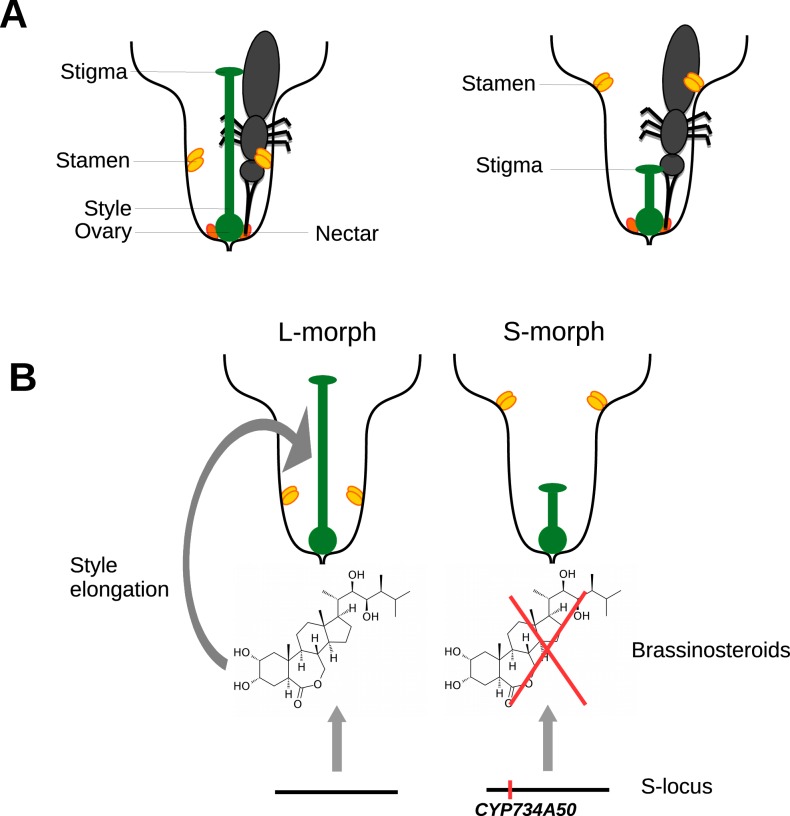
Heterostyly in primroses. (**A**) Schematic diagrams of a long-styled (left) and short-styled flower (right) from a distylous species such as *Primula vulgaris*. When an insect enters a long-styled flower it would pick up pollen mainly on its head. If this insect then visits a short-styled flower, the stigma would pick up the pollen from the insect’s head. Meanwhile, the high stamens would deposit fresh pollen on to the insect’s abdomen for effective transfer to the stigmas of long-styled flowers. (**B**) Huu et al. identified CYP730A50 as a duplicated gene that alters style length in *Primula* flowers. Flowers without CYP730A50 (left) produce brassinosteroids, which cause the cells in the style to elongate and produce a long style. On the other hand, flowers with this gene (right) in the S-locus suppress the production of brassinosteroids, which leads to a short style.

Charles Darwin was fascinated by heterostyly (Darwin, 1877) and we now know that it is caused by the expression of several genes that are inherited together as a single genetic unit (locus). However, working out how these genes influence the development of flowers has been something of a holy grail in the field. Now, in eLife, Michael Lenhard at the University of Potsdam and colleagues – including Cuong Nguyen Huu as first author – have identified the gene that is responsible for the different lengths of styles in primroses, and shown how it works (Huu et al., 2016).

Huu et al. found that a gene called CYP734A50 is only present in short-styled flowers in several species of primrose, and estimates of transcript read coverage suggest that only one copy of the gene is present in these flowers. CYP734A50 encodes an enzyme that degrades plant hormones called brassinosteroids, which are known to promote cell elongation (Ohnishi et al., 2006). Huu et al. found that a particular brassinosteroid, castasterone, is present at higher levels in long styles than in short styles, and that treating short styles with another brassinosteroid increased style length (Figure 1B). Moreover, virus-induced silencing of CYP734A50 in young plants with short styles resulted in the production of flowers with longer styles.

It has been thought for a long time, based on theoretical reasoning and comparative analysis, that the first mutation in the evolution of heterostyly was a mutation that reduced style length, and that the expression of this mutated gene should be dominant over the expression of its homologous gene at the style-length locus (Lloyd and Webb, 1992a; 1992b). The results of Huu et al. are consistent with the idea that the expression of the CYP734A50 gene in short-styled individuals is genetically dominant. But how did this dominance come about?

Huu et al. show that CYP734A50 is the result of the duplication of a gene that degrades brassinosteroids more generally. The duplication event occurred early in the evolution of the *Primula* genus, and the duplicated copy then evolved to be expressed only in styles. The presence or absence of the duplication has been maintained in the genus by a process called negative frequency-dependent selection, whereby the fitness of a trait (e.g. short styles) increases if it becomes rare, thus preventing its loss.

In his short autobiography, Darwin reflected that "I do not think anything in my scientific life has given me so much satisfaction as making out the meaning of the structure of heterostylous flowers". Like so much of his work, the hypothesis that he put forward has stood the test of time and remains the favoured explanation for how and why heterostyly evolved (Barrett, 2002). By identifying the gene that controls style length, the work of Huu et al. is an important step towards understanding the underlying mechanisms involved in heterostyly. A future challenge is to understand the evolutionary history of the other genes involved in heterostyly and to work out what roles they play in flower development.
